# Adsorption Studies on the Removal of Anionic and Cationic Dyes from Aqueous Solutions Using Discarded Masks and Lignin

**DOI:** 10.3390/molecules28083349

**Published:** 2023-04-10

**Authors:** Penghui Li, Chi Yang, Yanting Wang, Wanting Su, Yumeng Wei, Wenjuan Wu

**Affiliations:** 1 Jiangsu Co-Innovation Center of Efficient Processing and Utilization of Forest Resources, Nanjing Forestry University, Nanjing 210037, China; 2 College of Light Industry and Food Engineering, Nanjing Forestry University, Nanjing 210037, China

**Keywords:** lignin, face mask, carbon material, adsorption, dyes

## Abstract

The carbon materials derived from discarded masks and lignin are used as adsorbent to remove two types of reactive dyes present in textile wastewater: anionic and cationic. This paper introduces the results of batch experiments where Congo red (CR) and Malachite green (MG) are removed from wastewater onto the carbon material. The relationship between adsorption time, initial concentration, temperature and pH value of reactive dyes was investigated by batch experiments. It is discovered that pH 5.0–7.0 leads to the maximum effectiveness of CR and MG removal. The equilibrium adsorption capacities of CR and MG are found to be 232.02 and 352.11 mg/g, respectively. The adsorption processes of CR and MG are consistent with the Freundlich and Langmuir adsorption models, respectively. The thermodynamic processing of the adsorption data reveals the exothermic properties of the adsorption of both dyes. The results show that the dye uptake processes follow secondary kinetics. The primary adsorption mechanisms of MG and CR dyes on sulfonated discarded masks and alkaline lignin (DMAL) include pore filling, electrostatic attraction, π-π interactions and the synergistic interactions between the sulphate and the dyes. The synthesized DMAL with high adsorption efficiency is promising as an effective recyclable adsorbent for adsorbing dyes, especially MG dyes, from wastewater.

## 1. Introduction

Nowadays, organic dyes are usually applied to the production of colored paper, wood, textiles and food materials, etc. The production process in these industries often leads to the release of dye effluent in large amounts, which puts the ecosystem and human health at risk [1]. Dyes are one of the major water pollutants. The typical dyes include azo, anthraquinone, indigo and phthalocyanine derivatives, all of which are persistent due to their complex structures and xenobiotic properties [2,3]. As a common anionic benzidine azo dye, Congo red (CR) can produce toxic substances in an anaerobic environment and cause pollution when discharged into water [4]. Malachite green (MG) is a cationic triarylmethane dye as well as one of the most used dyes for coloring. It requires a type of therapeutic agent to treat fungal and bacterial infections in fish and is applicable as an antiseptic to treat wounds and ulcers. However, MG has the potential to cause cancer, mutations, teratogenicity and respiratory toxicity. It is thus important to remove MG dyes from wastewater prior to their discharge [5]. Usually, among various organic dyes, synthetic dyes (e.g., CR, MG, rhodamine B and methyl orange) are known as one of the major pollutants due to their cost reduction, easy access and effectiveness. This is attributed to the lack of suitable substitutes, the difficulty in controlled release, their latent toxicity, as well as mutagenic and carcinogenic activities [6,7]. In addition, many dyes have been found to be carcinogenic, which has an adverse effect on the development of plants and animals in lakes, rivers and so on. The color of dyes decreases the penetration of light in water bodies and affects photosynthetic activity, which makes it attractive to explore the recovery of organic dyes from water [8]. Reportedly, there are over 100,000 known varieties of commercial dyes in the global field, the production capacity of which exceeds 700,000 tons annually [9]. Therefore, it is imperative to achieve the purification of dye wastewater for water resources conservation.

Nowadays, many in-depth studies have been conducted on various treatment techniques for dye removal, such as biodegradation, membrane filtration, adsorption, oxidation, flocculation precipitation, ozone oxidation, chemical precipitation, catalytic degradation, etc. [10,11,12]. However, most of the above methods are subject to limitations and are incapable of achieving the complete removal of dyes from wastewater. Adsorption is a promising technology that can be applied to the removal of dyes [13,14]. So far, plenty of research has been conducted to promote the development of various adsorbents for wastewater treatment. Activated carbon, metal oxides, functionalized cellulose, molecular sieves, metal-organic skeletons and porous resins have been studied and applied to the removal of organic dye pollutants [15,16,17,18,19,20,21]. At present, activated carbon adsorption material is a kind of physical adsorption method in industry which is applied widely and efficiently, and has distinctive profitable properties for dye separation, such as high surface area, uniform pore size, good mechanical and thermal stability [22]. Furthermore, the adsorption performance can be significantly improved by doping sulphur atoms into carbon-based materials, and it has a significant effect on increasing the number of active sites of the adsorbent [23]. The process of water purification using cheaper absorbents is now more economically viable [12,24,25,26].

Lignin is one of the main components of lignocellulosic biomass, as well as the second most abundant biopolymer on Earth [27]. Production of lignin, a by-product of the process, has now reached 50 million tons per year. With in emerging bioethanol market, lignin emissions are expected to further increase [28]. In the paper-making industry, more than 90% of lignin is expelled or burned off as black liquor due to its stubborn structure, which not only causes the waste of resources but also results in environmental pollution [29]. Due to the aromatic ring network structure of lignin, it is easy to form graphite-like domains during the activation process, which makes the carbon materials obtained usable as battery electrodes. In addition to low-grade absorbents, it is another ideal precursor for lignin to produce high-quality carbon materials. Compared to coal-derived activated carbons, lignin-based activated carbons have a reduced carbon footprint [30]. Among the various preparation methods of lignin-based activated carbon, KOH activation is commonly used to produce the activated carbon with high specific surface area in the most efficient way [31].

Since December 2019, the outbreak of COVID-19 has attracted attention worldwide [32]. According to statistics, the quantity of masks discarded per month reaches 129 billion around the world, which means there are about 3 million masks discarded every single minute [33]. However, their recycling is infeasible due to the high biohazard potential. The carbonization of polymeric waste provides a promising solution to this problem. The effective use of surgical mask waste enables the recovery of the waste and gives full play to its potential without causing the depletion of non-renewable resources [34]. Discarded masks can be transformed into valuable carbon material by converting them into activated carbon via the capture of CO_2_ [35]. Sreńscek-Nazzal et al. [36] investigated the production of carbon-based electrode materials from hazardous surgical mask waste through KOH activation and carbonization. The obtained micro-mesoporous materials have a specific surface area of 460–969 m^2^/g and a total pore volume of 0.311–0.635 cm^3^/g.

As a promising approach to utilizing the energy of these wastes and their organic value, co-processing technologies include co-gasification, co-combustion, co-combustion and co-pyrolysis [37]. In this study, biomass plastic materials are mixed at a certain ratio (1:1, *w*/*w*). The discarded masks are sulfonated with concentrated sulfuric acid and subsequently physically mixed with alkaline lignin, which is mixed and activated by adding NaOH solution. After calcination, the porous carbon structures is formed with hollow spheres and applied to remove both types of dyes: CR and MG. SEM, TEM, TG, BET, XRD and XPS are used in combination to characterize the structure, morphology and phase changes of resulting adsorbents. Furthermore, the effects of adsorbent dosage, adsorption time, pH value and initial dye concentration on the adsorption performance are investigated. On this basis, the adsorption mechanism followed by the prepared materials on CR and MG is discussed in-depth.

## 2. Results and Discussion

### 2.1. Characterization of the Prepared Adsorbent

**Figure 1:**
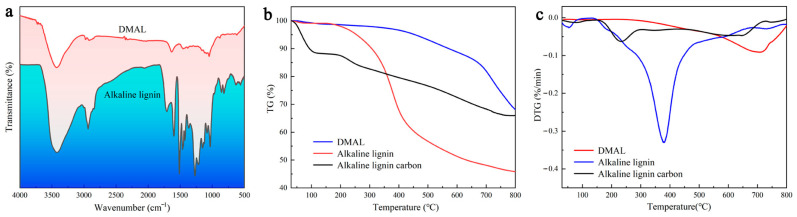
(**a**) IR spectrum of DMAL with alkaline lignin, (**b**) TG curve of DMAL with alkaline lignin, (**c**) DTG curve of DMAL with alkaline lignin.

As shown in Figure 1a, there are more functional groups of alkaline lignin but fewer functional groups of the resulting co-pyrolysis products, and the characteristic bands associated with lignin either almost disappear or further diminish. The peaks at 620, 853, and 1041 cm^−1^ indicate not only the weakening of sulphonic acid groups but also the shift of the spectra of the high-temperature carbonized sulfonated face mask and alkaline lignin. DMAL fails to detect a band at 1198 cm^−1^ that is characteristic of the sulphonate group [38]. The C-SOx-C (x = 2, 3, 4) group is reported to be unstable and prone to decomposition at high temperatures, but the thiophene structure C-S-C is more stable than C-SOx-C (x = 2, 3) [39,40]. The decomposition temperature of the alkaline lignin is relatively low, while the pyrolysis temperature required by the mixture of discarded masks and alkaline lignin is high (710 °C) (Figure 1b). The high thermal weight loss of alkaline lignin facilitates the generation of activated carbon, while the addition of masks improves the thermal stability of the co-pyrolysis products and enhances the adsorption capacity of the activated carbon products [41,42]. The DTG curve for the pyrolysis of the mixture shows a peak when the temperature is between 700 °C and 730 °C, which is associated with chain-breaking reactions and carbon formation (Figure 1c). However, lignin decomposition occurs in the temperature range of 370 °C to 400 °C. In the presence of discarded masks, the temperature required to decompose the mixture shows an upward trend. Apparently, the addition of discarded masks causes the peak temperature of lignin to rise [43].

**Figure 2:**
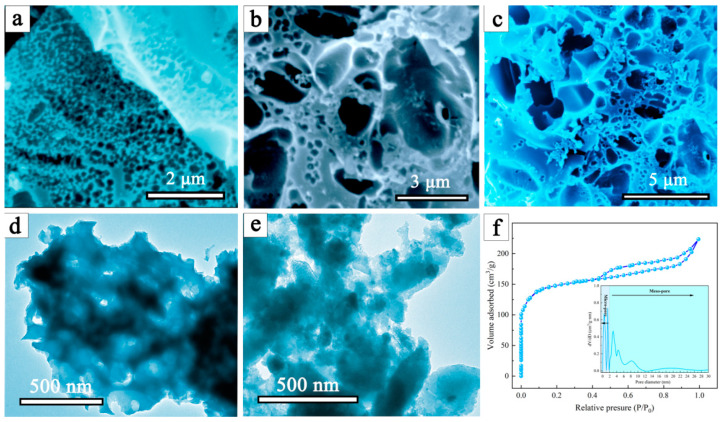
(**a**–**c**) SEM images of DMAL, (**d**) TEM image of alkaline lignin carbon, (**e**) TEM image of DMAL, and (**f**) nitrogen adsorption-desorption isotherms of the DMAL carbon material, inset shows the pore size distribution of DMAL.

Figure 2a–c shows the SEM images of a waste mouthpiece with an alkaline lignin-derived carbon material, from which a distinct pore structure (abundant macro- and mesopores) can be easily found. Thus, during the charring process, the interaction between the lignin and the polypropylene in the mask allows the creation of porous structures. According to the TEM images shown in Figure 2d,e, the alkali lignin carbon material clearly agglomerate, and the DMAL shows a highly heterogeneous surface with extensive irregularities. As indicated by nitrogen adsorption-desorption isotherms (Figure 2f), the specific surface area of DMAL is 659 m^2^/g, much higher compared to pure lignin-based carbon materials (183 m^2^/g). It was calculated that the total pore volume according to N_2_ adsorption at P/P_0_ = 0.98 is 0.51 cm^3^/g. According to the IUPAC classification, the isotherms of these materials are mixed in form between II and IV. In addition, the pore size distribution (inset of Figure 2f) reveals that the pore sizes of DMAL concentrate in the micro- and mesoporous regions. Taking into account the SEM analysis shown in Figure 2a–c, DMAL is confirmed to have plenty of macro/meso/micro pores. Notably, these porous structures facilitate the trapping of CR/MG and their rapid diffusion onto the DMAL surface [44]. Therefore, DMAL is applicable to remove large molecular pollutants such as CR and MG dyes from aqueous solutions.

**Figure 3:**
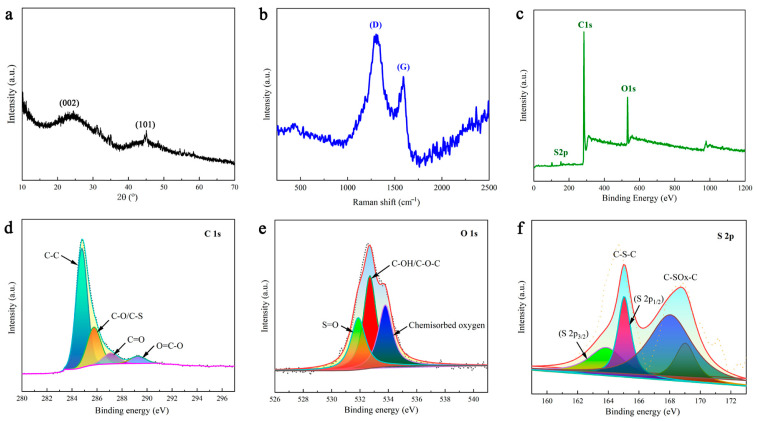
(**a**) XRD patterns, (**b**) raman spectra and (**c**) high resolution XPS spectra of full spectrum (**d**) C 1s, (**e**) O 1s and (**f**) S 2p from DMAL.

Figure 3a shows the XRD pattern of DMAL. These broad peaks at approximately 24° and 43° correspond to the (002) and (101) reflections of the disordered carbon layer, respectively. In addition, the high intensity in the low-angle region is suspected to result from the presence of numerous micropores in DMAL [45]. The broad peak suggests the formation of a predominantly amorphous carbon structure, which is basically consistent with the conclusions drawn by others [35,36]. Raman spectroscopy was applied to measure the degree of crystallinity of DMAL. As shown in Figure 3b, the peaks at 1587 cm^−1^ (G-band) and 1324 cm^−1^ (D-band) are ascribed to the graphitic carbon and lattice-defective carbon in DMAL, respectively [46]. Figure 3c shows that DMAL contains a significant amount of carbon and oxygen, along with a small amount of sulphur. According to the data obtained from the XPS fit, the mass content of individual atoms is 85.10% for carbon, 13.45% for oxygen and 1.45% for sulphur. Figure 3d shows the high-resolution C 1s spectrum. The peak at 284.5 eV is formed by the sp^2^ hybridized carbon atoms, indicating that the majority of carbon atoms exist in the graphite layer. Three peaks at about 285.2, 286.2 and 289.2 eV are attributable to the sp^2^ carbons connected to other atoms, such as C-O and/or C-S, C=O, and O=C-O [38]. As shown in Figure 3e, DMAL contains a great quantity of oxygen-containing functional groups. The binding energies of about 513.5, 532.5 and 533.6 eV correspond to S=O groups, C-OH and/or C-O-C groups and chemisorbed oxygen, respectively. The peak of 536.4 eV in the O 2p spectrum indicates the presence of sulphate material [38]. Figure 3f shows the S 2p XPS spectra of DMAL, whereas Figure 2f shows the S 2p XPS spectra of DMAL. There are four peaks shown by the spectrum of the lower sulphur content DMAL carbonized sample. The two peaks at about 163.8 and 165.0 eV correspond respectively to S 2p_3/2_ and S 2p_1/2_ for thiophene sulphide (-C-S-C-) and/or -C-S-C- sulphide bridges, while the other two peaks at about 168.0 and 169.0 eV are attributed to oxygenated sulphur groups -C-SOx-C- (x = 2, 3, 4), such as sulphone, sulphonate and sulphate groups. The peaks of the sulfonic acid group as shown in the XPS results after carbonization are consistent with the IR spectrum [38].

### 2.2. Optimization of Adsorption Conditions

**Figure 4:**
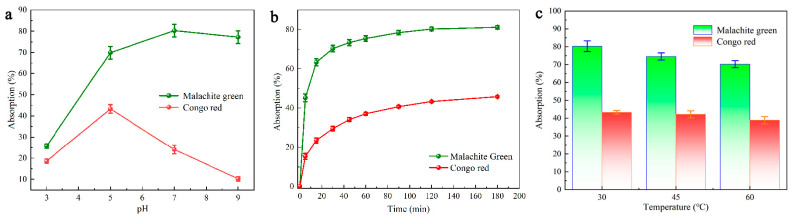
CR and MG adsorption on (**a**) effect of pH, (**b**) effect of time, (**c**) effect of temperature.

Reportedly, it is common for electrostatic interaction to occur between the dye molecule and the adsorbent, and that the initial pH is a determinant for the adsorption performance [47]. When the pH is set to two or raised to the range of 10–12, the CR solution undergoes a color change. For this reason, the pH was maintained in the range of 4–9 for the CR adsorption experiments (dye concentration based on the absorbance of each dye, i.e., 100 mg/L CR and 25 mg/L MG) [48]. As shown in Figure 4a, the adsorption of CR by the carbon material DMAL continues to decrease in the pH range 4–9, while MG keeps increasing. As can be seen from the above, DMAL has the best adsorption performance for MG when pH = 7, and for CR when pH = 5.

Figure 4b shows the effect of stirring time on dye removal at fixed concentrations and optimal pH values for each dye. DMAL removes a considerable amount of dye during the first 120 min of contact and causes the amount of DMAL adsorbed within 120–180 min to stabilize. In addition, adsorption studies were performed on two dyes to investigate the effect of temperature on adsorption. Given the optimum pH and an adsorption time of 120 min, the adsorption of the two dyes was measured at three different temperatures (30, 45 and 60 °C). According to Figure 4c, the adsorption of both MG and CR by DMAL diminishes as the solution temperature increases. Therefore, the absorption of the two dyes under study is considered to be inherently exothermic.

### 2.3. Isotherm Modeling

**Figure 5:**
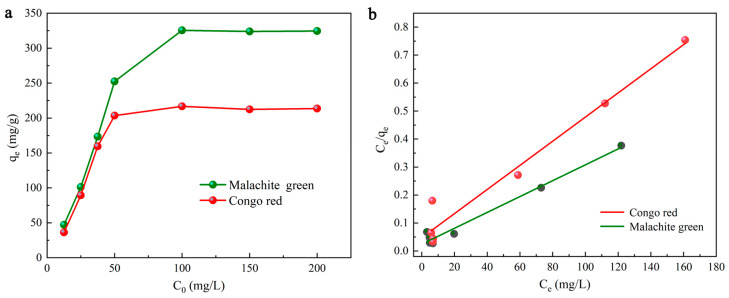
The effects of the initial dyes concentration (**a**), the Langmuir isotherm model (**b**).

The adsorption equilibrium data apply to the Langmuir and Freundlich isotherms, both of which are the most used two-parameter equations and usually expressed as [27]:

(1) $$\frac{C_{e}}{q_{e}}=\frac{C_{e}}{q_{max}}+\frac{1}{K_{L}q_{max}}$$

where the Langmuir constant *K_L_* is defined as the attraction of the active site of DMAL [49]. When the Langmuir model is used, a curve can be obtained, as shown in Figure 5a,b. With *C_e_* on the horizontal axis and *C_e_*/*q_e_* on the vertical axis, a straight line can be fitted to find *q_max_* (mg/g) and *K_L_* (L/g) by slope and intercept [50]. The *q_max_* (mg/g) for MG is calculated to be 352.11, 346.76 and 342.43 mg/g at different temperatures (303.15 K, 318.15 K, 333.15 K), while that for CR is calculated to be 232.02, 231.45 and 227.85 mg/g at different temperatures (303.15 K, 318.15 K and 333.15 K), and other parameters are also listed in Table 1. The correlation coefficient (*R*^2^ > 0.982) indicates that the results of adsorption experiments are consistent with those of the Langmuir model.

The Freundlich model is used due to the adsorption occurring at heterogeneous adsorption sites on the surface of the medium and in the presence of interactions between dye molecules. The logarithmic form of the Freundlich model is presented as follows [50]:

(2) $$logq_{e}=logK_{F}+\frac{1}{n}logC_{e}$$

where *K_F_* (L/g) represents the adsorption capacity, which is closely related to the adsorption affinity of the carbon material, and n refers to the favorable factor for adsorption. In general, *1/n* is smaller when the adsorbent shows a greater effectiveness of adsorption. It has been widely known that adsorption occurs easily when 0.1 ≤ 1/*n* ≤ 0.5, but it becomes difficult when 1/*n* ≥ 2 [49]. *K_F_* and n are determined through the logarithmic relationship between *C_e_* and *q_e_*. The constants of the fitted isotherms based on the Freundlich model can be found in Table 1. As suggested by the correlation coefficient (*R*^2^), the adsorption of MG and CR by DMAL contradicts the Freundlich model. The correlation coefficients for the Langmuir isotherm Freundlich isotherm are listed in Table 2. Through a numerical comparison shown in Table 2, it can be concluded that the Langmuir model fits better than the Freundlich model. The data for the Dubinin-Radushkevich isotherm model, Temkin isotherm model, can be found in Table S1.

### 2.4. Thermodynamic Study

**Figure 6:**
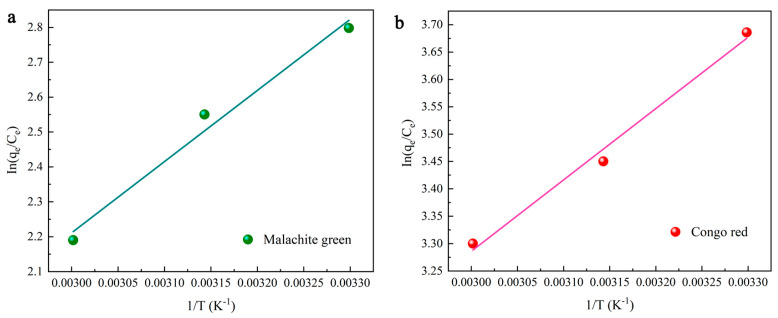
Thermodynamic study on the adsorption of MG (**a**) and CR (**b**) dyes by DMAL.

With an increase in temperature, the adsorption capacity declines (Figure 4c). The thermodynamic parameters, including free energy (Δ*G*^0^), enthalpy (Δ*H*^0^) and entropy (ΔS^0^), can be obtained using Equations (3)–(5). These parameters are required to evaluate the thermodynamic properties of the adsorption of MG and CR on DMAL [63]. The calculation process for these parameters is shown in Figure 6.

(3) $$K_{e}^{0}=K_{L}{\left[absorbate\right]}^{0}/\gamma$$

(4) $$∆G=-RT{InK}_{e}^{0}$$

(5) $${InK}_{e}^{0}=-{∆H}^{0}/RT+{∆S}^{0}/R$$

where *K* is the equilibrium constant, *K*_e_^0^ (dimensionless) is the adsorption equilibrium constant, *K*_L_ (L/mol) is the Langmuir equilibrium constant, [absorbate]^0^ (mol/L) is the standard concentration of the adsorbate, γ (dimensionless) is the activity coefficient, and *R* is the gas constant. The values of the above thermodynamic parameters are listed in Table 3. The negative values of free energy indicate the viability of each dye for adsorption onto DMAL. In addition, higher negative values are found as the temperature gradually increases from 30 to 60°C and decreases. This is because temperature rise suppresses dye adsorption. The negative value comes from the increase in energy released when the dye interacts with the adsorbent surface. In fact, this result suggests the formation of exothermic hydrogen bond and the thermal effect of adsorption in the process of DMAL adsorption. The thermodynamic evaluation confirms the exothermic nature of the adsorption process. When the temperature rises from 303.15 K to 333.15 K for MG adsorption, Δ*G*^0^ increases from −6.50 to −5.46 kJ/mol, indicating that the adsorption process is promoted by a slightly higher temperature than the ambient temperature. The negative value of the entropy change (Δ*S*^0^ = −34.48 kJ/mol) indicates a random decrease in adsorption occurring at the solid-liquid interface.

### 2.5. Kinetic Study

As an essential and positive factor affecting the adsorption process, adsorption kinetics can be measured to evaluate the effectiveness of adsorption and the various processes of the adsorption reaction. Pseudo-first-order kinetics are based on the assumption that the post-adsorption mechanism tends to show physisorption properties [63]. It can be explained by the following equation:

(6) $$\log\left(q_{e}-q\right)=\log q_{e}-\frac{k_{1}}{2.303}t$$

where *k*_1_ represents the pseudo-first-order rate constant whose value is calculated using the slope of the linear plot between time (*t*) and log (*q_e_*-*q*).

The second-order kinetic model is expressed as [66]:

(7) $$\frac{t}{q}=\frac{1}{k_{2}q_{e}^{2}}+\frac{1}{q_{e}}t$$

where *k*_2_ (g/mg·min) represents the rate constant for secondary adsorption, *q* indicates the measure of dye adsorbed at time *t* (mg/g), and *q_e_* denotes the quantity of dye adsorbed at equilibrium (mg/g). The applicability of the kinetic model was determined by comparing the phase relation values. The individual parameters of the second-order kinetic model are listed in Table 4, which shows that the fitted correlation coefficients for CR and MG exceed 0.99. Furthermore, the calculated results agree well with the experimental data. It is suggested that the second-order kinetic model fits better in describing the adsorption of DMAL.

It is also possible for the intra-particle diffusion of the dye MG with CR to occur on the carbon material DMAL. In this study, an intra-particle diffusion model is applied to simulate this process. This model is expressed as [50]:

(8) $$q=k_{id}t^{1/2}+C_{i}$$

where *k_id_* (mg/g·min^1/2^) represents the kinetic constant for intraparticle diffusion, and *C_i_* denotes the intercept of stage i, which is defined as the effect of the boundary layer on molecular diffusion. The above two parameters, *k_id_* and *C_i_*, are obtained from the slope and intercept of the fitted line of *q* versus *t*^1/2^ (Table 3). In the intraparticle diffusion model, if the line obtained by fitting q to *t*^1/2^ fails to pass through the origin, it implies that the adsorption process is not the only barrier to intraparticle diffusion.

**Figure 7:**
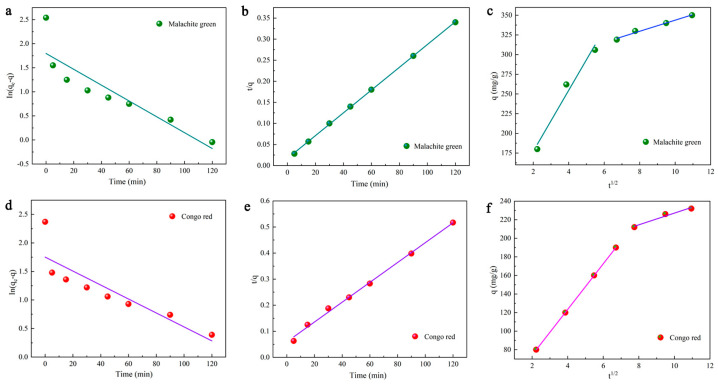
Adsorption kinetic studies of dye adsorption onto DMAL, pseudo-first-order model of MG (**a**) and CR (**d**); pseudo-second-order model of MG (**b**) and CR (**e**); intragranular model of MG (**c**) and CR (**f**).

As shown clearly in Figure 7, the fitted line has two components. In the first part, the slope is steeper and the change is faster, which proves that the outer surface of DMAL is absorbed faster. The second part has a gentle slope, and the adsorption rate becomes low, which may result from the intraparticle (pore) diffusion of the DMAL carbon material [50]. The *k_id1_* (38.80 mg/g·min^1/2^) of MG is much higher than that of *k_id2_* (7.05 mg/g·min^1/2^), and that (24.58 mg/g·min^1/2^) of CR is higher compared to *k_id2_* (6.30 mg/g·min^1/2^). It denotes that external diffusion is the main contributor to the adsorption dynamics. Moreover, it can be found from Figure 7c,f that this fitted line consisting of two segments fails to pass through the (0,0) of the coordinates. That is to say, there are other steps involved in the adsorption process of DMAL. In summary, the adsorption of CR and MG by DMAL is a highly complex process [50,63,67,68].

### 2.6. Comparison with Other Adsorbents

A comparison was made between the maximum adsorption capacity of DMAL for MG and CR in this study and other adsorbent materials in literature. According to Table 2, the DMAL material synthesized in this study shows a larger specific surface area and higher adsorption capacity than other reported adsorbents. Therefore, DMAL is considered a promising adsorbent for organic dye removal.

## 3. Experimental

### 3.1. Materials

**Figure 8:**
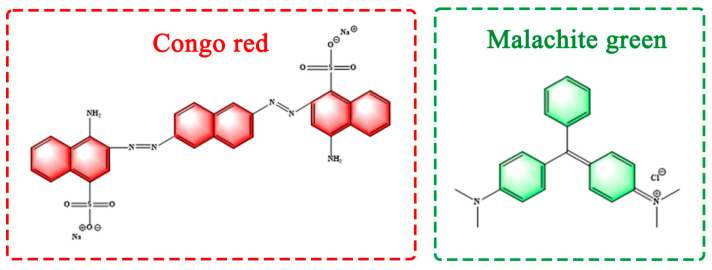
Structural formulae of CR and MG.

The commercially available reactive dyes CR and MG were purchased from Nanjing Chemical Reagent Co., Ltd. (China). Figure 8 shows the structure of these two dyes. The masks were obtained from discarded masks collected in our laboratory and placed in an oven at 120 °C to kill bacteria before the experiment was performed. The alkaline lignin was sourced from a paper mill in Jiangsu Province, China. Deionized water was used as the solution to simulate the wastewater. The maximum absorption wavelengths were determined. Solution of the two different anionic and cationic dyes, CR and MG, were prepared at different concentration gradients. The perceived color of the chemicals was directly affected by the absorption or reflectance in the visible range, and the maximum absorption wavelength of the dyes was determined by scanning the full UV-vis spectrum of CR and MG.

**Figure 9:**
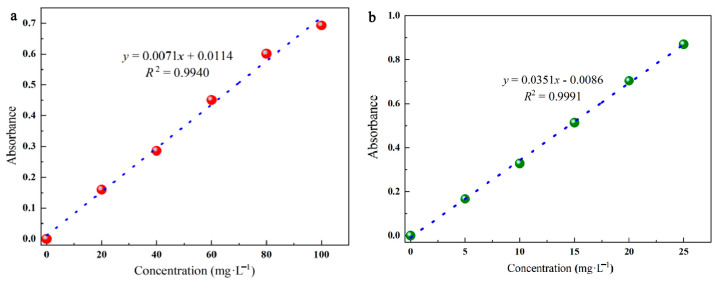
Equation of the fitted standard curve, CR (**a**) MG (**b**).

The standard curves were created using the following process. CR and MG were obtained in different concentration gradients, and their absorbance at the maximum absorption wavelength was measured at each concentration. As shown in Table 5, the linear relationship between the absorbance and the concentration was fitted, while the correlation coefficient *R*^2^ was applied to determine the goodness of the fitted relationship (0.99 or above). (The fitted standard curve is shown in Figure 9).

### 3.2. Materials Synthesis

The feedstock is present as black carbonaceous powder particles. To begin with, the samples of the discarded masks were treated with concentrated sulphuric acid for sulphonation. Then, they were washed with water to neutralize and then dried. The alkaline lignin was mixed with the dried masks and activated with KOH at an impregnation ratio of 1:1. Afterwards, the dried samples were heated by a tube furnace in a nitrogen atmosphere at 750 °C for 2 h. Finally, the black product was obtained, thoroughly cleaned with 1.0 M HCl and deionized water to remove inorganic impurities, and then dried at 105 °C for 24 h. The samples of masks sulfonated with alkaline lignin were obtained and labeled as DMAL. A reserve desiccator was used to keep the product in a vacuum until use.

### 3.3. Material Characterization

The TGA209 F1 Thermal Gravimetric Analyzer from NETZSCH, Germany was used to analyze the thermal stability of discarded masks, lignin carbon materials and DMAL at a temperature ranging from 30 to 800 °C and a heating rate of 10 °C/min under the protection of high-purity nitrogen. A Quanta 20 field emission scanning electron microscope purchased from Hitachi, Japan, was used to examine the microscopic morphological characteristics of the DMAL carbon material. The Brunauer-Emmett-Teller (BET) specific surface area was measured against nitrogen adsorption at 77 K on a surface area using a porosity analyzer (Micromeritics ASAP 2460, Norcross, GA, USA). The surface chemical composition of the samples, including the surface elemental composition and peaks of C (C 1s), O (O 1s), and S (S 2p), were determined by an AXIS Ultra DLD (Shimadzu, Japan) X-ray photoelectron spectrometer (XPS). Samples were detected at concentrations greater than 0.1% and depths less than 10 nm. The crystal structure of the polymers was analyzed using an Ultima IV combination-type multifunctional horizontal X-ray diffractometer.

### 3.4. Adsorption Test

Adsorption was determined by the batch method to facilitate the assessment of those parameters affecting the adsorption process. We use different types and concentrations of dyes to simulate wastewater. A set amount of DMAL (0.02 g) was added to 50 mL of different concentrations of CR/MG solution, contained in a 50 mL conical flask. Then, the solution was stirred continuously at a constant temperature. After an equilibration time (initially determined), the sample was removed. Spectrophotometric method was used to determine the effectiveness of dye removal at the maximum absorbance (for CR at 499 nm and MG at 620 nm). The adsorbed solution was collected at predetermined time intervals (5, 15, 30, 45, 60, 90, 120, and 180 min) and the dye content was determined. The influence of solution pH was analyzed by dye adsorption experiments at pH ranging from 3.0 to 9.0. To explore the impact of temperature on the experimental results, adsorption was conducted at different temperatures (30, 45, and 60 °C). The Langmuir and Freundlich isothermal models were used to study the adsorption equilibrium. In the same way, the kinetic data of the first and second-order rate equations were tested to fit the appropriate rate equation. The final results were obtained by averaging the results of three repeated measurements.

The adsorption capacity (*q*, mg/g) is expressed as follows:

(9) $$q=(C_{0}-C_{t})\times \frac{V}{W}$$

where *C*_0_ and *C_t_* (mg/L) represent the initial and final (after equilibrium is reached) concentrations of the dye, *V* (L) indicates the volume of adsorbate, and *W* (g) denotes the weight of the adsorbent.

The absorption and adsorption rate of unit mass DMAL under equilibrium state are as follows: *q_e_* (mg/g) and absorption (%):

(10) $$q=(C_{0}-C_{t})\times \frac{V}{W}$$

(11) $$Adsorption(\%)=\frac{\left(C_{0}-C_{e}\right)}{C_{0}}\times 100$$

## 4. Conclusions

It is essential to remove dyes from wastewater effectively and consistently to mitigate their negative impact on the environment. In this study, DMAL carbon material is verified to be an effective, high-efficiency and inexpensive adsorbent to remove reactive dyes from wastewater. Through adsorption tests, the optimal pH for adsorption is found to be weakly acidic and neutral. It is suspected that the high adsorption capacity of DMAL for dyes is related to the highly porous structure of the carbon material and the functional groups of the dyes. As revealed by the fitted isotherms for the adsorption of MG and CR dyes, the curves are consistent with the Langmuir isotherm model. We performed bulk adsorption experiments, aided by the Langmuir adsorption model, and obtained a CR adsorption capacity of 232.02 mg/g for DMAL and 352.11 mg/g for MG. Furthermore, thermodynamic parameters indicate that the physical adsorption of MG and CR molecules on the carbon material DMAL is inherently spontaneous and exothermic, with the adsorption effect decreasing with increasing temperature. The fitted kinetic curves of DMAL for the adsorption of CR and MG dyes are more suited for the pseudo-second-order model, which is in accordance with the fitted results obtained for the thermodynamic parameters. The Langmuir model is suitable, and the adsorption is confirmed as secondary kinetic. In conclusion, DMAL carbon materials are applicable as an alternative to those expensive adsorbents for textile dye wastewater treatment. The production and use of DMAL not only treat waste pulp and paper residues but also offer a new approach to the reuse of waste masks.

## Figures and Tables

**Table 1:** Langmuir and Freundlich adsorption isotherms constants for the adsorption of both reactive dyes on DMAL.

Dyes	Temperature (°C)	Langmuir Constant			Freundlich Constant		
		*q_max_* (mg/g)	*K_L_* (L/mg)	*R* ^2^	*K_F_* (L/g)	*1/n*	*R* ^2^
MG	30	352.11	0.12	0.985	154.76	0.25	0.51
	45	346.76	0.11	0.984	147.23	0.26	0.48
	60	342.43	0.09	0.982	142.47	0.29	0.41
CR	30	232.02	0.09	0.990	108.98	0.05	0.28
	45	231.45	0.08	0.987	105.43	0.05	0.39
	60	227.85	0.06	0.983	99.48	0.08	0.32

**Table 2:** Maximum adsorption capacities of dyes onto DMAL different adsorbents.

Adsorbents	Dyes	Adsorption Capacity	Reference
Zn(OH)_2_-NP-AC	Sunset yellow	115	[51]
ZnO-NRs-AC	Bromocresol green	58	[52]
Al-CNTs-2.0	Methyl orange	70	[53]
AC-Al	Methylene blue	182	[54]
Pine cone biochar	Methylene blue	106	[55]
Sargassum fusiforme activated carbon	Congo red	234	[56]
H_2_SO_4_ modified celery residue	Congo red	239	[57]
ZnO-modified SiO_2_ NPs	Congo red	83	[58]
Carbon composite lignin	Congo red	199	[59]
CuO-NPs-AC	Malachite green	213	[60]
Apricot stones AC	Malachite green	88	[61]
Putrescible vegetable waste AC	Malachite green	31	[62]
Pinus roxburghii cone AC	Malachite green	250	[63]
Corn stalk and walnut shell mix-based AC	Malachite green	195	[64]
Fe_3_O_4_-carbon microspheres	Malachite green	469	[65]
DMAL	Congo red	232	This work
	Malachite green	352	

**Table 3:** Thermodynamic parameters for the adsorption of two reactive dyes on DMAL.

Dyes	Temperature (°C)	Δ*G*^0^ (kJ/mol)	Δ*H*^0^ (kJ/mol)	Δ*S*^0^ (J/mol·K)
MG	30	−6.50	−16.95	−34.48
	45	−5.98		
	60	−5.46		
CR	30	−9.26	−10.83	−5.18
	45	−9.18		
	60	−9.10		

**Table 4:** Adsorption kinetics constant and parameter values.

Kinetic Model	Parameters	MG	CR
Pseudo-first order	*k*_1_	0.038	0.028
	*q_e_*	62.72	56.51
	*R*_2_	0.777	0.739
Pseudo-second order	*k*_1_	0.0004	0.0002
	*q_e_*	370.37	263.16
	*R* ^2^	0.999	0.996
Intraparticle diffusion	*k_id1_*	38.80	24.58
	*C*	99.54	25.07
	*R*_1_^2^	0.972	0.999
	*k_id2_*	7.05	6.30
	*C*	273.27	164.07
	*R*_1_^2^	0.986	0.938

**Table 5:** Basic parameters of the two organic dyes.

Name	CR	MG
Molecular formula	C_32_H_22_N_6_Na_2_O_6_S_2_	C_23_H_25_N_2_Cl
Molecular weight	697	365
Type	Anionic	Cationic
Maximum absorption	499	620

## Data Availability

Data sharing is not applicable to this article as no datasets were generated or analyzed during the current study.

## Supplementary Materials

The following supporting information can be downloaded at: https://www.mdpi.com/article/10.3390/molecules28083349/s1, Table S1: Temkin and D-R adsorption isotherms constants for the adsorption of both reactive dyes on DMAL.

## Sample Availability

Samples of the DMAL are available from the authors.
