# Supplementary material for: Adsorption Studies on the Removal of Anionic and Cationic Dyes from Aqueous Solutions Using Discarded Masks and Lignin
Source: Molecules. 2023 Apr 10;28(8):3349. doi: 10.3390/molecules28083349 (PMC10143327; doi:10.3390/molecules28083349)
Supplement: Supplementary file 1 [file molecules-28-03349-s001.zip › molecules-2317771-supplementary.pdf]

Supplementary Data for the article

Adsorption studies on the removal of anionic and cationic dyes from aqueous solutions using discarded masks and lignin

Penghui Li[1,2], Chi Yang[2], Yanting Wang[2], Wanting Su[2], Yumeng Wei[2], Wenjuan Wu[1,2*]
[1] Jiangsu Co-Innovation Center of Efficient Processing and Utilization of Forest Resources, Nanjing Forestry University, Nanjing 210037, China;
[2] College of Light Industry and Food Engineering, Nanjing Forestry University, Nanjing, 210037, China;

Table S1. Temkin and D-R adsorption isotherms constants for the adsorption of both reactive dyes on DMAL

Dyes	Temperature (°C)	Temkin			D-R			
		A (mg/L)	*B*	R^2	$q_{m,D-R}$ *(mg/g)*	*β* *(mol²/KJ²)* $\times 10^{-8}$	*E(KJ/mol)* $\times 10^3$	R^2
MG	30	13.34	67.49	0.711	281.34	0.218	1.514	0.156
CR	30	89.34	32.77	0.467	175.45	5.434	3.033	0.108

Temkin adsorption isotherm model is based on the interaction between adsorbent and adsorbate which assumes that the adsorption heat decreases linearly with the increase of the degree of adsorption process, the linear expression is as follows [63]:

$$q_e = BIn_A + BInC_e \tag{1}$$

where, C_e (mg/L) is the equilibrium concentration, q_e (mg/g) is the equilibrium adsorption capacity, A (mg/L) is the equilibrium constant related to binding energy, and B is the Temkin constant related to the adsorption heat.

Dubinin-Radushkevich (D-R) adsorption isotherm model does not require the above ideal assumption which is based on the Polanyi potential energy theory. The adsorption space of adsorbent surface is certain, and there is a temperature independent adsorption potential at each point of the adsorption space, the linear equation is as follows [63]:

$$Inq_e = -\beta\varepsilon^2 + Inq_{m,D-R} \tag{2}$$

where, $q_{m,D-R}$ (mg/g) is the theoretical maximum adsorption capacity of D-R adsorption isotherm model, β (mol^2/KJ^2) is the D-R model constant related to the adsorption energy, Ɛ is Polanyi adsorption potential. among them,

$$\varepsilon = RTIn(\frac{C_e+1}{C_e}) \tag{3}$$

where, R is the universal gas constant, T (K) is the thermodynamic temperature.

The average free energy E (KJ/mol) of the adsorption process can be calculated by the β constant of the D-R model, the formula is as follows:

$$E = \frac{1}{\sqrt{2\beta}} \quad (4)$$
